# Supplementary material for: Aperiodic 1/f noise drives ripple activity in humans
Source: Nat Commun. 2026 Jan 17;17:746. doi: 10.1038/s41467-026-68404-5 (PMC12820272; doi:10.1038/s41467-026-68404-5)
Supplement: Supplementary file 1 — Supplementary Information [file 41467_2026_68404_MOESM1_ESM.pdf]

## Supplementary Information for:

# Aperiodic 1/f noise drives ripple activity in humans

Frank J. van Schalkwijk[1,*] and Randolph F. Helfrich[2,3,4,*]

[1] Hertie-Institute for Clinical Brain Research, Center for Neurology, University Hospital Tübingen, Tübingen, Germany.

[2] Department of Psychology, Yale University, New Haven, CT, USA.

[3] Wu Tsai Institute, Yale University, New Haven, CT, USA.

[4] Interdisciplinary Neuroscience Program, Yale University, New Haven, CT, USA.

***Corresponding authors**

Frank J. van Schalkwijk: frankvanschalkwijk@gmail.com

Randolph F. Helfrich: randolph.helfrich@gmail.com

**Supplementary Note**

**Ripple detection on simulated data with variable 1/f characteristics per detector.** Replication of ripple detection, demonstrating how the noise profile of each methodology affects ripple detection on simulated data with varying exponents (Supplementary Fig. 1).

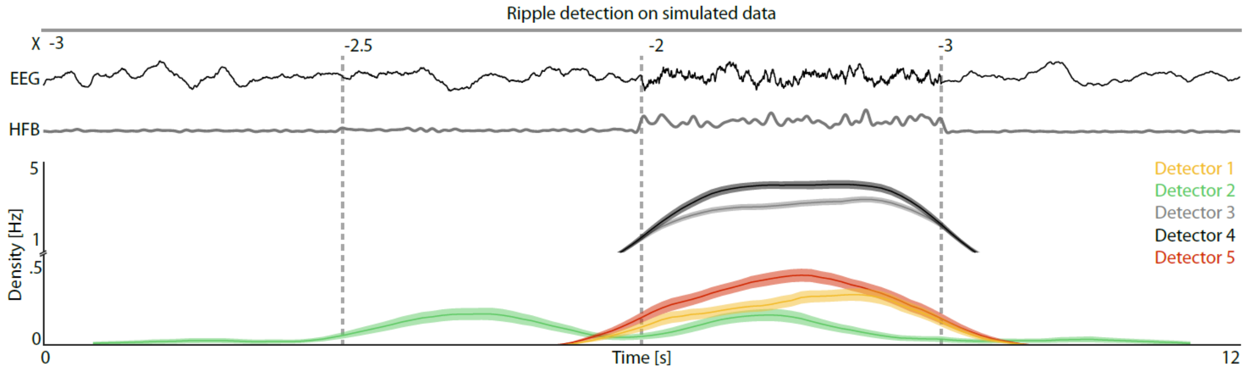


**Supplementary Figure 1 | Detecting ripple oscillations in variable 1/f noise simulations.** Demonstration of how the noise profile of each detector affects ripple detection on simulated data with variable 1/f characteristics. Upper row: Simulated signal with varying exponents (black) per 3 second segments. Middle row: Ripple-band activity (grey line; 80-120 Hz; z-normalized). Bottom row: Ripple density (Hz) per detector across identical simulated datasets and iterations (100 iterations; mean ± SEM). Source data are provided as Source Data file.

**Comparing model fits to estimate spectral characteristics and noise floor for ripple detection.** As no consensus has been established with regard to the optimal frequency range for spectral estimation, we repeated the analyses for the sleep data (Fig. 2) using different frequency ranges for spectral parameterization (1-40 Hz; 20-45 Hz; 30-100 Hz; and 2-128 Hz). All models utilized a linear fit (aperiodic mode = fixed).

Model fits showed high goodness-of-fit estimates ($R^2 \geq .97$), wherein the best fit was observed when the full spectrum was considered (1-40 Hz: $R^2 = .9830 \pm .0004$, mean ± SEM; 20-45 Hz: $R^2 = .9817 \pm .0008$; 30-100 Hz: $R^2 = .9846 \pm .0013$; 2-128 Hz: $R^2 = .9908 \pm .0002$). Spectral exponents from the original fit (20-45 Hz) were significantly correlated with the 1-40 Hz range (all $p \leq .0001$; rho = .09 ± .05 mean ± SEM), 30-100 Hz range (all $p \leq .0001$; rho = .36 ± .04), and the 2-128 Hz range (all $p \leq .0004$; rho = .25 ± .05).

Although all model fits differentiate between sleep states (Supplementary Fig. 2a), the 2-128 Hz range did not capture the regional differences observed for the narrower frequency range and showed an overall narrower distribution of spectral exponents across the night (Supplementary Fig. 2b) as also demonstrated recently [1]. Note that all models can be employed to estimate a noise floor for ripple detection (Supplementary Fig. 2b), albeit that the narrower distribution for the 2-128 Hz range results in the fact that all detected ripples fall within the noise floor boundaries. Combined, these simulations reveal a general pattern where ripple detection on experimental data has a wider distribution of exponent values as compared to simulated data.

In sum, our original approach (linear FOOOF model fit between 20-45 Hz) provides a very high goodness-of-fit ($R^2 = .9817 \pm .0008$; mean ± SEM), omits the spectral knees in low and high frequency ranges, avoids line noise or notch filtering artifacts, delineates regional differences, and can be successfully utilized to identify the noise floor for ripple detection.

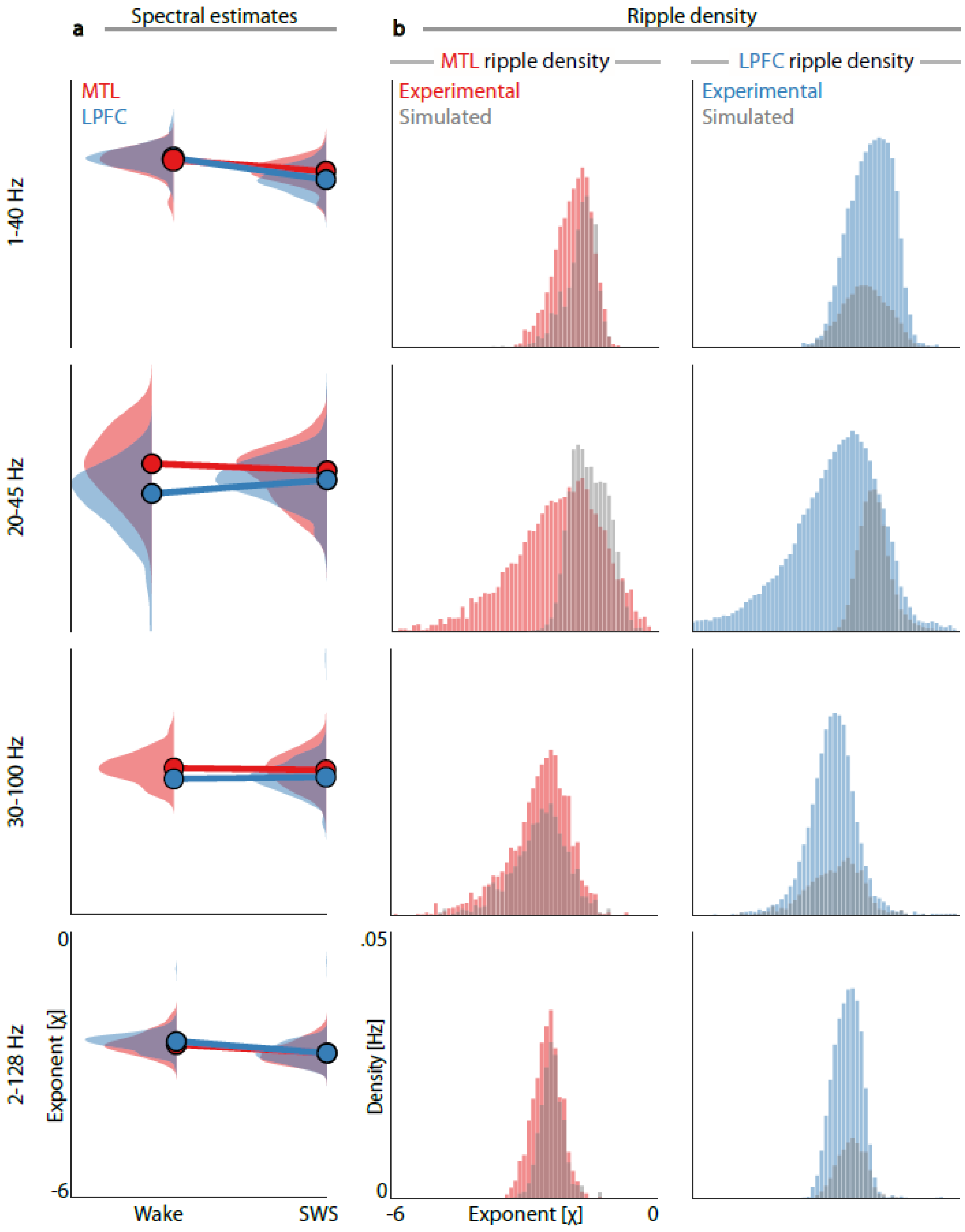


**Supplementary Figure 2 | State- and regional differences for spectral exponent values depends on the model fit. a** Group level (N=14) spectral exponents (y-axis) as a function of state (x-axis) and region (medial temporal lobe [MTL], $N_{electrodes}$=82, red; lateral prefrontal cortex [LPFC], $N_{electrodes}$=339, blue). Data are displayed as probability density functions. Model fits within the 20-45 Hz frequency range successfully differentiate between cognitive states and additionally capture regional differences. In contrast, a model fit across the entire spectrum (2-128 Hz) only differentiates between states. **b** Distribution of ripple density (y-axis) as a function of the spectral exponent (x-axis) in the MTL (red) and LPFC (blue). Simulation-based inference of ripple density (superimposed grey distribution) can be established regardless of the model's frequency range. Note how the distribution of spectral exponents is narrower for the model fit across the entire spectrum (Bottom) as compared to the alternative fits (Top & Middle), resulting in all events falling within the noise floor boundaries. Source data are provided as Source Data file.

**1/f noise characteristics severely impact ripple detection for all detectors.** To determine if the relation between the spectral exponent and ripple detection is dependent on the detection algorithm, we repeated this analysis for all detection algorithms. In a first step, we analyzed ripple density as a function of the spectral exponent (from -6.0 to 0, in 0.1 steps) for electrodes in the MTL and LPFC for observed and surrogate data (Supplementary Fig. 3a). For MTL ripples, we observed a broad distribution across many spectral exponents (Supplementary Fig. 3a Left), whereas ripples detected on 1/f noise-matched surrogated fell within a narrower, but substantially overlapping range of exponents. Note that the distributions for observed data all show a distinct peak at a spectral exponent of -2 for MTL electrodes and -2.5 for LPFC electrodes, whereas the distribution of detected ripples from the surrogate data varied as a function of detection algorithm (c.f., Fig. 1d). Similar patterns were observed for electrodes in the LPFC (Supplementary Fig. 3a Right).

To quantify these observations, we determined ripple density as a function of region and state for both observed and surrogate data. We observed different effects across the five detection algorithms. For the observed data recorded from the MTL (Supplementary Fig. 3b Left), ripple density from wakefulness to SWS was found to significantly decrease (detector 1: $p$ = .0001, [$CI_{95}$] = [-.001, -.003], $t_{162}$ = -3.90, $\beta$ = -.006; detector 5: $p$ < .0001, [$CI_{95}$] = [-.014, -.007], $t_{162}$ = -6.29, $\beta$ = -.01; LME), significant increase (detector 2: $p$ < .0001, [$CI_{95}$] = [.013, .022], $t_{162}$ = 7.45, $\beta$ = .017; detector 4: $p$ = .0383, [$CI_{95}$] = [.001, .046], $t_{162}$ = 2.09, $\beta$ = .024, LME), or remain unaffected by sleep stage (detector 3: $p$ = .2807, [$CI_{95}$] = [-.07, .02], $t_{676}$ = -1.08, $\beta$ = -.02; LME). For the surrogate data, we found significant decreases in ripple density from wakefulness to SWS for four out of five detectors (detector 1: $p$ < .0001, [$CI_{95}$] = [-.02, -.01], $t_{162}$ = -5.77, $\beta$ = -.017; detector 3: $p$ < .0001, [$CI_{95}$] = [-.56, -.22], $t_{162}$ = -4.53, $\beta$ = -.39; detector 4: $p$ = .0095, [$CI_{95}$] = [-.10, -.015], $t_{162}$ = -2.62, $\beta$ = -.059; detector 5: $p$ < .0001, [$CI_{95}$] = [-.03, -.01], $t_{162}$ = -5.25, $\beta$ = -.02; LME), whereas a significant increase was observed for detector 2 ($p$ = .0339, [$CI_{95}$] = [.0003, .0086], $t_{162}$ = 2.14, $\beta$ = .004; LME).

Changes in ripple density from wakefulness to SWS were similarly affected by detection algorithm for data recorded from the LPFC (Supplementary Fig. 3b Right). Ripple density from wakefulness to SWS was found to significantly decrease (detector 1: $p$ = .0012, [$CI_{95}$] = [-.004, -.001], $t_{676}$ = -3.25, $\beta$ = -.003; detector 3: $p$ < .0001, [$CI_{95}$] = [-.06, -.02], $t_{676}$ = -4.12, $\beta$ = -.04; detector 5: $p$ < .0001, [$CI_{95}$] = [-.011, -.007], $t_{676}$ = -9.67 $\beta$ = -.01; LME), significant increase (detector 2: $p$ < .0001, [$CI_{95}$] = [.020, .024], $t_{676}$ = 19.80, $\beta$ = .022; LME), or remain unaffected by sleep stage (detector 4: $p$ = .2392, [$CI_{95}$] = [-.007, .026], $t_{676}$ = 1.18, $\beta$ = .01; LME). For the surrogate data, we found significant increases in ripple density from wakefulness to SWS for all detectors (detector 1: $p$ < .0001, [$CI_{95}$] = [.005, .009], $t_{676}$ = 7.93, $\beta$ = .007; detector 2: $p$ < .0001, [$CI_{95}$] = [.003, .005], $t_{676}$ = 6.91, $\beta$ = .004; detector 3: $p$ < .0001, [$CI_{95}$] = [.42, .62], $t_{676}$ = 10.50, $\beta$ = .52; detector 4: $p$ < .0001, [$CI_{95}$] = [.22, .30], $t_{676}$ = 11.96, $\beta$ = .26; detector 5: $p$ < .0001, [$CI_{95}$] = [.011, .016], $t_{676}$ = 10.05, $\beta$ = .014; LME).

To illustrate and summarize these results, we calculated the percent overlap of observed ripples relative to the surrogate distribution (Supplementary Fig. 3c). In the MTL, we observed that the proportion of ripples falling within the noise floor significantly decreased from wakefulness to SWS (detector 1: $p$ < .0001, [$CI_{95}$] = [-37.09, -14.98], $t_{162}$ = -4.66, $\beta$ = -26.03; detector 2: $p$ = .0018, [$CI_{95}$] = [-13.51, -3.14], $t_{162}$ = -

3.17, $\beta$ = -8.33; detector 3: $p$ < .0001, $[CI_{95}]$ = [-42.88, -16.73], $t_{162}$ = -4.50, $\beta$ = -29.80; detector 4: $p$ < .0001, $[CI_{95}]$ = [-41.51, -18.56], $t_{162}$ = -5.17, $\beta$ = -30.04; detector 5: $p$ < .0001, $[CI_{95}]$ = [-37.41, -15.61], $t_{162}$ = -4.80, $\beta$ = -26.51; LME).

In contrast, the proportion of LPFC ripples falling within the noise floor significantly increased from wakefulness to SWS for four out of five detectors (detector 1: $p$ < .0001, $[CI_{95}]$ = [16.43, 23.69], $t_{676}$ = 10.86, $\beta$ = 20.06; detector 3: $p$ < .0001, $[CI_{95}]$ = [24.73, 35.71], $t_{676}$ = 10.81, $\beta$ = 30.22; detector 4: $p$ < .0001, $[CI_{95}]$ = [27.08, 37.91], $t_{676}$ = 11.78, $\beta$ = 32.49; detector 5: $p$ < .0001, $[CI_{95}]$ = [19.32, 26.32], $t_{676}$ = 12.80, $\beta$ = 22.82; LME), whereas a decrease was observed for detector 2 (detector 2: $p$ < .0001, $[CI_{95}]$ = [-20.13, -12.89], $t_{654}$ = -8.95, $\beta$ = -16.51; LME).

We consequently observed significant interaction effects between the two regions for all detectors (detector 1: $p$ < .0001, $[CI_{95}]$ = [36.95, 55.24], $t_{838}$ = 9.90, $\beta$ = 46.10; detector 2: $p$ = .0362, $[CI_{95}]$ = [-15.76, -.53], $t_{816}$ = -2.10, $\beta$ = -8.14; detector 3: $p$ < .0001, $[CI_{95}]$ = [47.16, 72.88], $t_{838}$ = 9.16, $\beta$ = 60.02; detector 4: $p$ < .0001, $[CI_{95}]$ = [50.71, 74.88], $t_{838}$ = 9.93, $\beta$ = 62.53; detector 5: $p$ < .0001, $[CI_{95}]$ = [40.44, 58.22], $t_{838}$ = 10.89, $\beta$ = 49.33; LME).

Collectively, this set of findings demonstrates that region- and state-specific differences in 1/f noise characteristics severely impact ripple detection for all detectors, especially if the detector noise susceptibility (Fig. 1d) peaks within the experimental condition as exemplified for MTL ripples. Albeit that all detectors are affected by the 1/f noise characteristics, state-specific changes in ripple density were not uniform across detection algorithms.

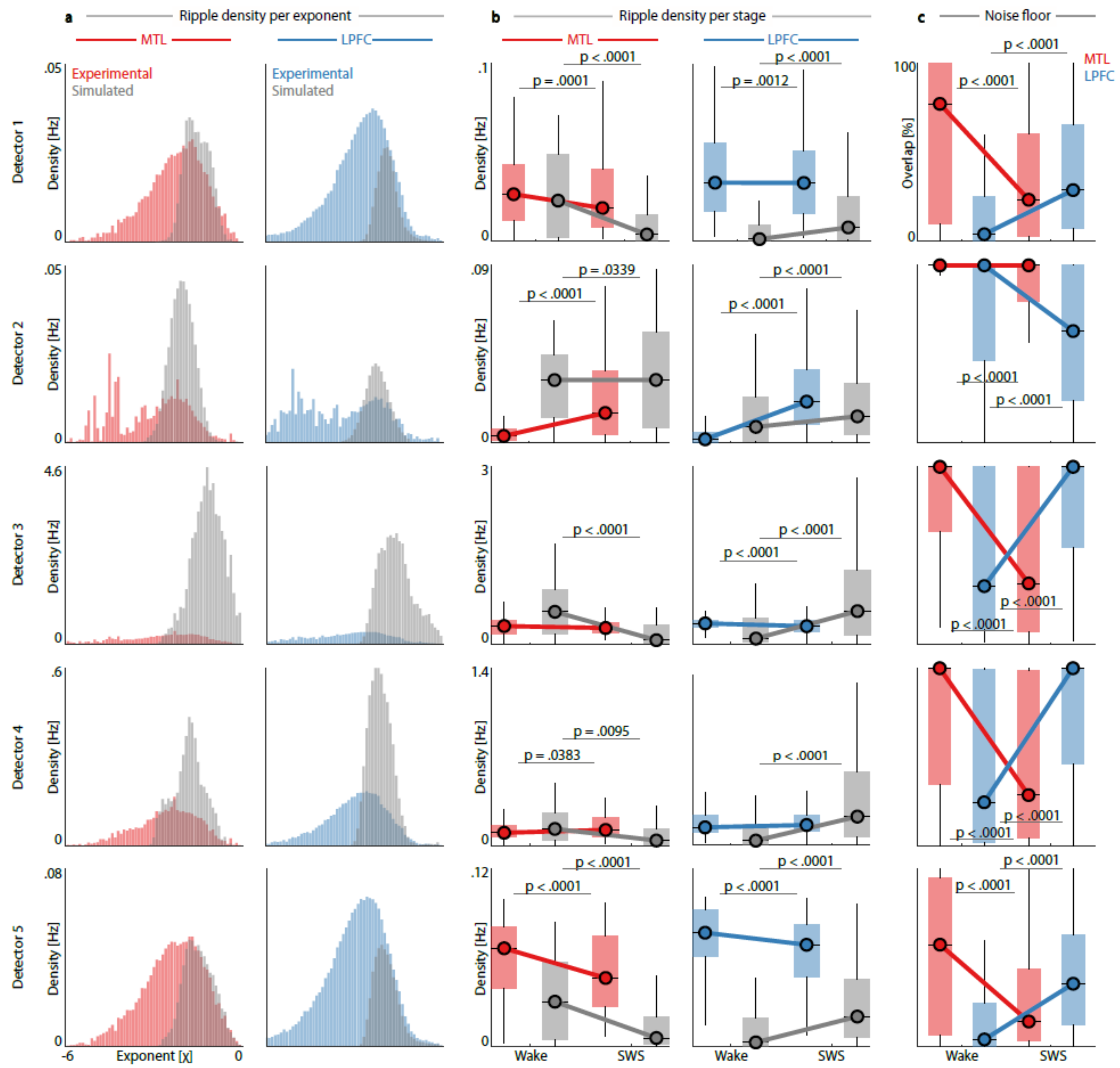


**Supplementary Figure 3 | 1/f noise characteristics variably explain region- and state-specific ripple density as a function of detection algorithm. a** Region- and state-specific relationship between noise and ripples on group level (N=14). *Left:* Distribution of ripple events (y-axis) as a function of the spectral exponent (x-axis) in the medial temporal lobe (MTL; $N_{electrodes}$=82, red) per detection algorithm, highlighting a clear relationship similar to the distribution reported in Figure 1d. Ripple detection in noise-matched epochs (superimposed grey distribution) highlights MTL ripples within the noise range and therefore, likely do not constitute genuine ripple events. *Right*: Distribution of ripple events relative to the spectral exponent in the lateral prefrontal cortex (LPFC; $N_{electrodes}$=339, blue; same conventions as in the left panels). **b** Mean event density per state. Left: We observed more ripples in the MTL during wakefulness than during SWS, as a direct result of higher spectral exponents during wakefulness (cf. top panel Fig. 2c). Given the overall lower exponents during SWS, it is more likely to detect true ripples during SWS than during wakefulness. Boxplots represent median (middle line), 25$^{th}$ and 75$^{th}$ percentiles (box), and extreme values. Right: Mean event density per state in the LPFC. Same conventions as in the left panels. **c** Percentage of ripples within the noise floor. Note that especially ripple detection in the MTL during wakefulness exhibit high percentages of false positives. Same conventions as in panel b. Source data are provided as Source Data file.

## Tasked-locked power spectra

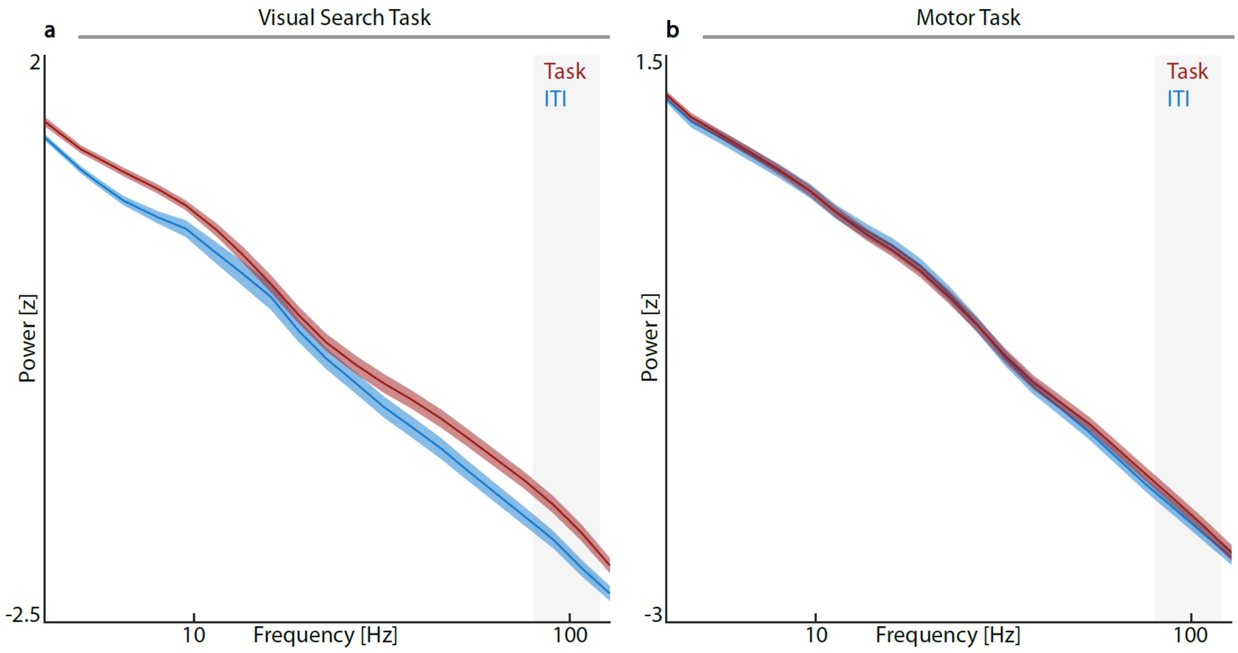


**Supplementary Figure 4 | Cognitive engagement increases the spectral exponent. a** Power spectrum (mean ± SEM) time-locked to trial execution of a Visual Search Task (Task) or inter-trial intervals (ITI) on group level (N=5; $N_{electrodes}$=34). Note the increase in high-frequency power during trial execution, which resulted from an increase of the spectral exponent (c.f., Fig. 3e). Gray shading indicates the ripple frequency range in humans (80-120 Hz). **b** Power spectrum of the Motor task and ITIs on group level (N=18; $N_{electrodes}$=99). Same conventions as in panel a. Again, note the increase in high-frequency power as a result of the spectral exponent increase (c.f., Fig. 3i). Source data are provided as Source Data file.

**Task-locked modulation of the spectral exponent is independent of frequency range.** To determine the robustness of our findings, we repeated spectral parameterization of our wake datasets (Fig. 3d, h Top) using multiple spectral fitting ranges (1-40 Hz; 20-45 Hz; 30-100 Hz; and 2-128 Hz). Again, condition-specific changes of the spectral exponent values were assessed.

For the Visual Search Task, we demonstrate that the spectral exponent increases during the task epoch as compared to inter-trial intervals (ITIs; Supplementary Fig. 5A). Within the time domain, the spectral exponent increased during task epochs as compared to the inter-trial interval (ITI) (Supplementary Fig. 5A). Statistical quantification was conducted at the pseudo-population level using RM-ANOVAs, and now includes frequency range as an additional factor, demonstrating significant main effects of condition (Supplementary Fig. 5B; $F_{2,66} = 7.82$, $p = .0085$, $\eta^2 = .19$, RM-ANOVA) and frequency range ($F_{3,99} = 21.73$, $p < .0001$, $\eta^2 = .40$, RM-ANOVA), as well as an interaction effect between condition and frequency range ($F_{6,198} = 4.92$, $p = .0114$, $\eta^2 = .13$, RM-ANOVA).

A qualitatively and quantitatively highly comparable set of findings was observed for the Motor Task. In the time domain, the spectral exponent increases during the task epoch as compared to inter-trial intervals (ITIs) (Supplementary Fig. 5C). Statistical quantifications demonstrate significant main effects of condition (Supplementary Fig. 5B; $F_{2,196} = 105.51$, $p < .0001$, $\eta^2 = .52$, RM-ANOVA) and frequency range ($F_{3,294} = 339.60$, $p < .0001$, $\eta^2 = .78$, RM-ANOVA), as well as an interaction effect between condition and frequency range ($F_{6,588} = 58.67$, $p < .0001$, $\eta^2 = .37$, RM-ANOVA).

In sum, these effects show that the spectral exponent is modulated by task condition irrespective of frequency range used for spectral decomposition. Yet, the magnitude of the effect differs between frequency ranges, and depends on the model parameters used for spectral parameterization. Specifically, the frequency range considered, as well as the inclusion of a spectral knee, may account for the current variability [2,3].

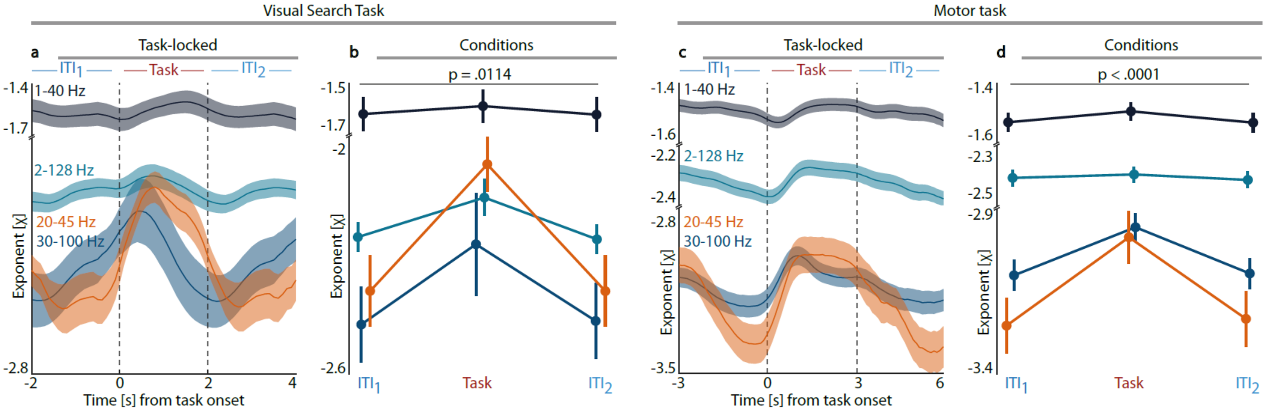


**Supplementary Figure 5 | Task-locked modulation of 1/f spectral exponent can be observed for multiple frequency range. a** Time-resolved spectral exponent values per frequency range (colored traces; mean ± SEM) during execution of a visual search task (N=5; $N_{electrodes}$=34). Note the modulation during task execution as compared to the pre and post inter-trial intervals (ITI) per frequency range considered for spectral parameterization. **b** Statistical quantification. The spectral exponent (median ± SEM) significantly increases during the task epoch as compared to the ITI ($F_{6,198} = 4.92$, $p = .0114$; RM-ANOVA). Same conventions as in panel a. Offset of the x-axis is for visualization purposes only. **c-d** Noise modulation during movement execution (N=18; $N_{electrodes}$=99), showing a significant increase during task as compared to ITI ($F_{6,588} = 58.67$, $p < .0001$; RM-ANOVA). Same conventions as in panels a-b. Source data are provided as Source Data file.

## Supplementary References


1. Lendner, J. D., Lin, J. J., Larsson, P. G. & Helfrich, R. F. Multiple intrinsic timescales govern distinct brain states in human sleep. *J. Neurosci.* **44**, e0171242024 (2024).
2. Donoghue, T. A Systematic Review of Aperiodic Neural Activity in Clinical Investigations. *European Journal of Neuroscience* **62**, e70255 (2025).
3. Ameen, M. S., Jacobs, J., Schabus, M., Hoedlmoser, K. & Donoghue, T. Temporally resolved analyses of aperiodic features track neural dynamics during sleep. *Commun Psychol* **3**, 160 (2025).
