# Supplementary material for: Aperiodic 1/f noise drives ripple activity in humans
Source: Nat Commun. 2026 Jan 17;17:746. doi: 10.1038/s41467-026-68404-5 (PMC12820272; doi:10.1038/s41467-026-68404-5)
Supplement: Supplementary file 2 — Reporting Summary [file 41467_2026_68404_MOESM2_ESM.pdf]

nature portfolio

Corresponding author(s): Frank J. van Schalkwijk and Randolph F. Helfrich

Last updated by author(s): Dec 18, 2025

Reporting Summary

Nature Portfolio wishes to improve the reproducibility of the work that we publish. This form provides structure for consistency and transparency in reporting. For further information on Nature Portfolio policies, see our Editorial Policies and the Editorial Policy Checklist.

Statistics

For all statistical analyses, confirm that the following items are present in the figure legend, table legend, main text, or Methods section.

n/a	Confirmed	
☐	☒	The exact sample size (*n*) for each experimental group/condition, given as a discrete number and unit of measurement
☐	☒	A statement on whether measurements were taken from distinct samples or whether the same sample was measured repeatedly
☐	☒	The statistical test(s) used AND whether they are one- or two-sided<br>*Only common tests should be described solely by name; describe more complex techniques in the Methods section.*
☐	☒	A description of all covariates tested
☐	☒	A description of any assumptions or corrections, such as tests of normality and adjustment for multiple comparisons
☐	☒	A full description of the statistical parameters including central tendency (e.g. means) or other basic estimates (e.g. regression coefficient) AND variation (e.g. standard deviation) or associated estimates of uncertainty (e.g. confidence intervals)
☐	☒	For null hypothesis testing, the test statistic (e.g. *F*, *t*, *r*) with confidence intervals, effect sizes, degrees of freedom and *P* value noted<br>*Give P values as exact values whenever suitable.*
☒	☐	For Bayesian analysis, information on the choice of priors and Markov chain Monte Carlo settings
☒	☐	For hierarchical and complex designs, identification of the appropriate level for tests and full reporting of outcomes
☐	☒	Estimates of effect sizes (e.g. Cohen's *d*, Pearson's *r*), indicating how they were calculated

Our web collection on statistics for biologists contains articles on many of the points above.

Software and code

Policy information about availability of computer code

Data collection	Intracranial EEG data were recorded at the University of California Irvine Medical Center, USA, using a Nihon Kohden recording system (model JE120A; 256-channels) at a sampling frequency of 5000 Hz and analog filter at 0.01 Hz.
Data analysis	All data preprocessing and analyses were conducted using Matlab R2021a (MathWorks Inc.). Data preprocessing, filtering, and segmentation were conducted using Fieldtrip (Oostenveld et al., 2011; fieldtrip-20241025) and EEGlab (Delorme & Makeig, 2004 eeglab2020_0) in addition to custom code.

For manuscripts utilizing custom algorithms or software that are central to the research but not yet described in published literature, software must be made available to editors and reviewers. We strongly encourage code deposition in a community repository (e.g. GitHub). See the Nature Portfolio guidelines for submitting code & software for further information.

Data

Policy information about availability of data

All manuscripts must include a data availability statement. This statement should provide the following information, where applicable:

- Accession codes, unique identifiers, or web links for publicly available datasets
- A description of any restrictions on data availability
- For clinical datasets or third party data, please ensure that the statement adheres to our policy

Source Data are provided with this paper. The electrophysiological task data are available at https://searchworks.stanford.edu/view/zk881ps0522.

Research involving human participants, their data, or biological material

Policy information about studies with human participants or human data. See also policy information about sex, gender (identity/presentation), and sexual orientation and race, ethnicity and racism.

Reporting on sex and gender	We included data from 14 patients with pharmacoresistant epilepsy (36.79 years ± 13.28, mean ± SD; range 19–58 years; 9 female). We had no a priori expectation of sex- or gender-related differences. We therefore did not conduct any sex- or gender-based analyses.
Reporting on race, ethnicity, or other socially relevant groupings	We had no a priori expectation of race, ethnicity, or other socially relevant differences. We therefore did not conduct any analyses based on race, ethnicity, or other socially relevant differences.
Population characteristics	We recorded data from 14 patients with pharmacoresistant epilepsy (36.79 years ± 13.28, mean ± SD; range 19–58 years; 9 female) undergoing invasive monitoring for seizure onset localization using intracranial depth electrodes. Medical diagnosis, medication type and dosage, as well as age of epilepsy onset were not considered as covariates in our analyses.
Recruitment	Patients undergoing invasive monitoring were recruited at the University of California Irvine Medical Center and included based on electrode coverage in the medial temporal lobe and prefrontal cortex. Patients were recorded on a per-case basis and underwent invasive electroencephalography based on clinical requirements.
Ethics oversight	Informed consent was obtained from all subjects prior to study participation. Data acquisition and analyses protocols were approved by the Institutional Review Board at the University of California, Irvine (protocol number: 2014–1522) and the Committee for Protection of Human Subjects at the University of California, Berkeley (Protocol number: 2010–02–783). The study was conducted in accordance with the 6th Declaration of Helsinki.

Note that full information on the approval of the study protocol must also be provided in the manuscript.

Field-specific reporting

Please select the one below that is the best fit for your research. If you are not sure, read the appropriate sections before making your selection.

- [x] Life sciences
- [ ] Behavioural & social sciences
- [ ] Ecological, evolutionary & environmental sciences

For a reference copy of the document with all sections, see nature.com/documents/nr-reporting-summary-flat.pdf

Life sciences study design

All studies must disclose on these points even when the disclosure is negative.

Sample size	Sample size calculation was not performed. Patients undergoing invasive monitoring were recruited at the University of California Irvine Medical Center and included based on electrode coverage in the medial temporal lobe and prefrontal cortex. We included 14 patients that were reported previously in van Schalkwijk et al., 2023, Progress in Neurobiology. We additionally analyzed two freely available datasets from Miller and colleagues (https://searchworks.stanford.edu/view/zk881ps0522), thus, included another 5 and 19 patients, respectively. This sample size is similar to comparable studies (e.g. Weber et al., 2023, Nat. Commun., n = 19; Schreiner et al., 2024, Nat. Commun., n = 10; Topalovic et al., 2023, Nat. Neuro., n = 12).
Data exclusions	For study 2, one subject was excluded from analyses because of a shorter trial length (2 s instead of 3 s duration).
Replication	Within-subject electrophysiological effects were replicated across different participants and studies.
Randomization	As our analyses considered simulated data, sleep recordings, and stimulus-evoked responses, no randomization was applied.
Blinding	Participants were not aware of the main hypotheses of the experiment. No group allocation was implemented during data collection and analysis. It was therefore not necessary to blind the experimenters to group allocation.

Reporting for specific materials, systems and methods

We require information from authors about some types of materials, experimental systems and methods used in many studies. Here, indicate whether each material, system or method listed is relevant to your study. If you are not sure if a list item applies to your research, read the appropriate section before selecting a response.

Materials & experimental systems

n/a	Involved in the study
☒	☐ Antibodies
☒	☐ Eukaryotic cell lines
☒	☐ Palaeontology and archaeology
☒	☐ Animals and other organisms
☒	☐ Clinical data
☒	☐ Dual use research of concern
☒	☐ Plants

Methods

n/a	Involved in the study
☒	☐ ChIP-seq
☒	☐ Flow cytometry
☒	☐ MRI-based neuroimaging

Plants

Seed stocks: n/a

Novel plant genotypes: n/a

Authentication: n/a
